# Pharmacokinetic/pharmacodynamic integration of tilmicosin against *Pasteurella multocida* in a piglet tissue cage model

**DOI:** 10.3389/fvets.2023.1260990

**Published:** 2023-09-04

**Authors:** Yuqin Chen, Xuan Ji, Suiling Zhang, Wenxiang Wang, Huilin Zhang, Huanzhong Ding

**Affiliations:** Guangdong Key Laboratory for Veterinary Drug Development and Safety Evaluation, College of Veterinary Medicine, South China Agricultural University, Guangzhou, China

**Keywords:** *Pasteurella multocida*, Tilmicosin, PK/PD relationship, tissue cage infection model, *in vivo*

## Abstract

Tilmicosin is a semi-synthetic macrolide for veterinary use with strong antibacterial effect on respiratory bacteria. In this study, the pharmacokinetic/pharmacodynamic (PK/PD) integration of tilmicosin against *Pasteurella multocida* (*P. multocida*) was evaluated by establishing a piglet tissue cage infection model. Concentration of tilmicosin and bacterial numbers of *P. multocida* in the tissue-cage fluid were monitered. After the population of *P. multocida* was equal to or greater than 10^7^ CFU/mL in a tissue cage, piglets received an oral administration of tilmicosin at a dose of 30, 40, 50, and 60 mg/kg b.w., once daily for 3 days, respectively. Bacteria were counted every 24 h after drug administration and at 48 and 72 h after the last administration. A sigmoidal E_max_ model was used to fit the relationship between PK/PD parameters and the antibacterial effect. AUC_24h_/MIC was the best PK/PD index that correlated with effectiveness of tilmicosin against *P. multocida*. The magnitude of AUC_24h_/MIC required for continuous 1/3-log, 1/2-log, and 3/4-log reductions were 19.65 h, 23.86 h, and 35.77 h, respectively, during each 24 h treatment period. In this study, when the dosage was >50 mg/kg, the AUC_24h_/MIC was still >35.77 h in the period of 24–48 h after the last administration due to the slow elimination, that is, tilmicosin exhibited a potent antibacterial effect against *P. multocida* after three successive daily administrations. The data provide meaningful guidance to optimize regimens of tilmicosin to treat respiratory tract infections caused by *P. multocida*.

## 1. Introduction

*Pasteurella multocida* (*P. multocida*) caused acute infectious diseases such as hemorrhagic septicemia or infectious pneumonia in various animals. The infection was often accompanied by the risk of other bacteria, causing porcine respiratory disease complex (PRDC). Multiple serotypes and complex typing make it difficult to prevent and control by vaccine (1). Tilmicosin is a macrolide synthesized from tylosin, which showed strong antibacterial activity against *Actinobacillus pleuropneumoniae*, *P. multocida*, and *Mycoplasma* (2–4).

The pharmacokinetics of tilmicosin was characterized by rapid absorption, slow elimination, and large apparent volume of distribution (V_d_). Futhermore, the higher concentration in lungs relative to plasma makes it a valuable treatment option for respiratory infections. According to Zhang et al. (5), the C_max_ of tilmicosin in lung tissues was 14.28 times higher than in plasma in chickens. In rabbits, the concentrations attained in lung tissue exceeded that in plasma by 7-fold after the second day of treatment (6). Foster et al. (7) determined that in calves, the concentration of tilmicosin in bronchoalveolar lavage samples exceeded plasma concentration by 776%. The absorption half-time (T_1/ka_) and elimination half-life (T_1/2kel_) of tilmicosin in healthy pigs were 2.27 h and 43.53 h after oral administration, respectively, and 1.64 h and 20.69 h in another study (8, 9). The V_d_ in pigs was 48.36 L/kg after a single oral administration and the V_d_ in both sheep and cattle were 25.0 L/kg when normalized for animal body weight, indicating the extensive tissue distribution of tilmicosin (10, 11).

Studies indicated that tilmicosin exhibited higher efficacy in treating bovine respiratory tract infection compared to oxytetracycline (12). Tilmicosin mixed feed was often used to treat and prevent respiratory diseases in pigs. However, irrational use of antimicrobials leads to severe acquired resistance of bacteria, resulting in treatment failure (13). PK/PD analysis could describe the relationship between the kinetics and the effect to establish rational dosage regimens of antimicrobial agents. PK/PD modeling in veterinary medicine can be used to define the relationship among concentrations, time, and therapeutic outcomes to more truly describe the dynamic relationship among drugs, hosts, and pathogens. Tissue cage model is a common method to study PK/PD *in vivo*. A tissue cage made of silicone tube, small volume, is suitable for animals with tight skin, mainly pigs. The model has been used to study the PK/PD integration of cefquinome and tulathromycin on *P. multocida in vivo* (14, 15). Therefore, it is of great significance to study the pharmacokinetics and pharmacodynamics of tilmicosin against *P. multocida* in pigs to ensure the rational use of drugs.

For pigs, it is difficult to obtain the drug concentrations and bacterial load at the target tissue in lungs of *P. multocida* in fection. The tissue cage model is an ideal model to study the relationship between PK/PD parameters and the antibacterial effect, which considers the interaction of the drug, host, and pathogen. The objective of the study was to determine the relationship between pharmacokinetic/pharmacodynamic indices and the antibacterial efficacy of tilmicosin against *P. multocida*. Additionally, the findings could inform the development of tilmicosin dosage regimens for treating respiratory illnesses caused by *P. multocida* infection.

## 2. Materials and methods

### 2.1. Bacterial strain, antimicrobials, and chemicals

Strain C44-15 (serovar D:7) of *P. multocida* was obtained from the China Institute of Veterinary Drug Control (Beijing, China). Timicosin phosphate with a purity of 80.4% (Tilmicosin) was provided by Guangdong Dahuanong Animal Health Products (Guangdong, China). Pentobarbital sodium was purchased from Xiangbo Biotechnology Co., Ltd. Procainamide hydrochloride was supplied by Shangdong Fangming pharmaceutical Co., Ltd. Tryptic soy broth (TSB) and tryptic soy agar (TSA) were purchased from Guangdong Huankai Microbial Technology (Guangzhou, China). Newborn bovine serum was supplied by Guangzhou Ruite Biotechnology (Guangzhou, China).

### 2.2. Animals and tissue-cage infection model

The study used 10 healthy crossbred piglets (Duroc × Landrace × Yorkshire) weighing 25–30 kg acquired from the Guangzhou Fine Breed Swine Farm. The piglets were given antibiotic-free feed and water over the course of the experiment. All the experimental procedures involving the animals were approved by the Committee on the Ethics of Animals of South China Agricultural University (approval number: 2018A001).

The specifications and surgical implantation procedures for the tissue cages (TCs) matched those described previously (16). The TCs were constructed from food-grade silicone tubes measuring 65 mm in length, 13 mm inner diameter, and 18 mm outer diameter. Twelve holes were made at each end of the tubes. The TCs were surgically implanted under general anesthesia induced by pentobarbital sodium and local anesthesia from procainamide hydrochloride. Sterile TCs were inserted subcutaneously on both sides of the pigs’ necks, positioned equidistantly between the jugular vein and spinal cord. Following surgery, the incision sites were treated with penicillin and tetracycline ointment for 3–5 days to prevent infection. After a 4-week recovery period, the TCs contained clear, yellow tissue cage fluid (TCF). The TCF was collected for bacteriological testing, and bacteria-free TCs were used for the experiment.

The sterile TCs were inoculated with 1 mL of a *P. multocida* suspension containing approximately 1.5 × 10^8^ CFU/mL. Following 48 h of incubation, 0.5 mL of TCF was extracted from each tissue cage to quantify the bacteria. TCs with bacterial counts around 10^7^ CFU/mL were selected for use in the subsequent experiment.

### 2.3. Minimum inhibitory concentration determination

The MIC of tilmicosin against *P. multocida* was ascertained using a microdilution technique following the guidelines of the Clinical and Laboratory Standards Institute (CLSI, 2013). The MIC value was derived from TSB supplemented with 5% calf serum and TCF cultures.

### 2.4. Experimental design and sample collection

Ten piglets were randomly divided into four treatment groups (two piglets and four tissues cages each group) and one control group (infected but not treatment). One male and one female pig were contained in each group. Each treatment group was administered orally with tilmicosin at the dosages of 30, 40, 50, and 60 mg/kg respectively, once a day for 3 days. The control group was treated with the same amount of normal saline.

For pharmacokinetics, around 0.5 mL of TCF was obtained at 1, 3, 6, 9, 12, and 24 h following each administration, and additional samples were collected at 48 and 72 h after the final dose. The samples underwent centrifugation at 3000 × *g* for 10 min to remove particulate matter and were kept frozen at −20°C until analyzed.

For pharmacodynamics, about 0.1 mL of TCF was collected for bacterial counting at 24 h after each administration and at 48 and 72 h after the last administration. The suspension was serially diluted 10-fold in saline, with 100 μL used initially. Subsequently, 20 μL of the dilutions were dispersed onto agar plates and incubated for 12 h to enumerate the bacterial population.

### 2.5. Quantification of tilmicosin in tissue cage fluid

The tilmicosin concentration was measured by high-performance liquid chromatography-tandem masss pectrometry (HPLC-MS/MS) using a system from Agilent Technologies (Santa Clara, CA, United States). Separation was achieved on a C_18_ column (150 mm × 2.0 mm, 5 μm; Phenomenex, Torrance, CA, United States). The mobile phase consisted of solution A (water with 0.1% formic acid, V/V) and solution B (acetonitrile) at a flow rate of 0.25 mL/min. The elution gradient was: 0–1.5 min, 10% B; 1.5–6 min, 95% B; 6–6.5 min, 5% B; 6.5–12.5 min, 5% B. The injection volume was 5 μL.

The samples were allowed to thaw to ambient temperature prior to analysis. Aliquots of 200 μL TCF were added to a 1.5 mL micro centrifuge tube and then equal volume acetonitrile was added to precipitate protein. After vortexing for 2 min, and centrifuging at 12000 × *g* for 10 min, the clear supernatant was filtered through a 0.22-μm nylon syringe filter (JinTeng Experiment Equipment Company) and then injected into an auto sampler vial. A calibration curve was established with tilmicosin concentrations (0.005–0.5 μg/mL). PK parameters were calculated using WinNonlin software version 6.1 (Pharsight, Mountain View, CA, United States).

### 2.6. Intergration and modeling of PK/PD

The PK/PD parameters were calculated based on the individual pharmacokinetics of each treated infected TCs: AUC_24h_/MIC (AUC_24h_, area under the concentration–time curve between 0 to 24 h after each dose), C_max_/MIC (C_max_, maximum concentration of tilmicosin after each dose), and %T > MIC (the percentage of time that tilmicosin concentration above MIC after each dosing interval). The relationship between the PK/PD indices and efficacy were evaluated using the sigmoidal maximum effect (E_max_) PD model. The formula was as follows:


$$E=E_{0}+\frac{\left(E_{\max}-E_{0}\right)\times C_{e}^{N}}{EC_{50}^{N}+C_{e}^{N}}$$

Where E represents the antibacterial effect, defined as the change in bacterial count (log_10_CFU/mL) over a 24 h dosing interval; E_0_ denotes the change of bacterial count in the control sample; E_max_ is the maximum antibacterial effect during the dosing interval; C_e_ signifies the value of a PK/PD index (C_max_/MIC, AUC_24h_/MIC, and %T > MIC); EC_50_ represents the PK/PD index value producing 50% of the maximal effect; and N is the Hill coefficient defining the slope of the effect curve.

## 3. Results

### 3.1. Minimum inhibitory concentration

The MIC values of tilmicosin against *P. multocida* (D:7) in both TCF and TSB broth were 0.25 μg/mL.

### 3.2. Pharmacokinetics of tilmicosin in the piglet tissue-cage infection model

**Figure 1:**
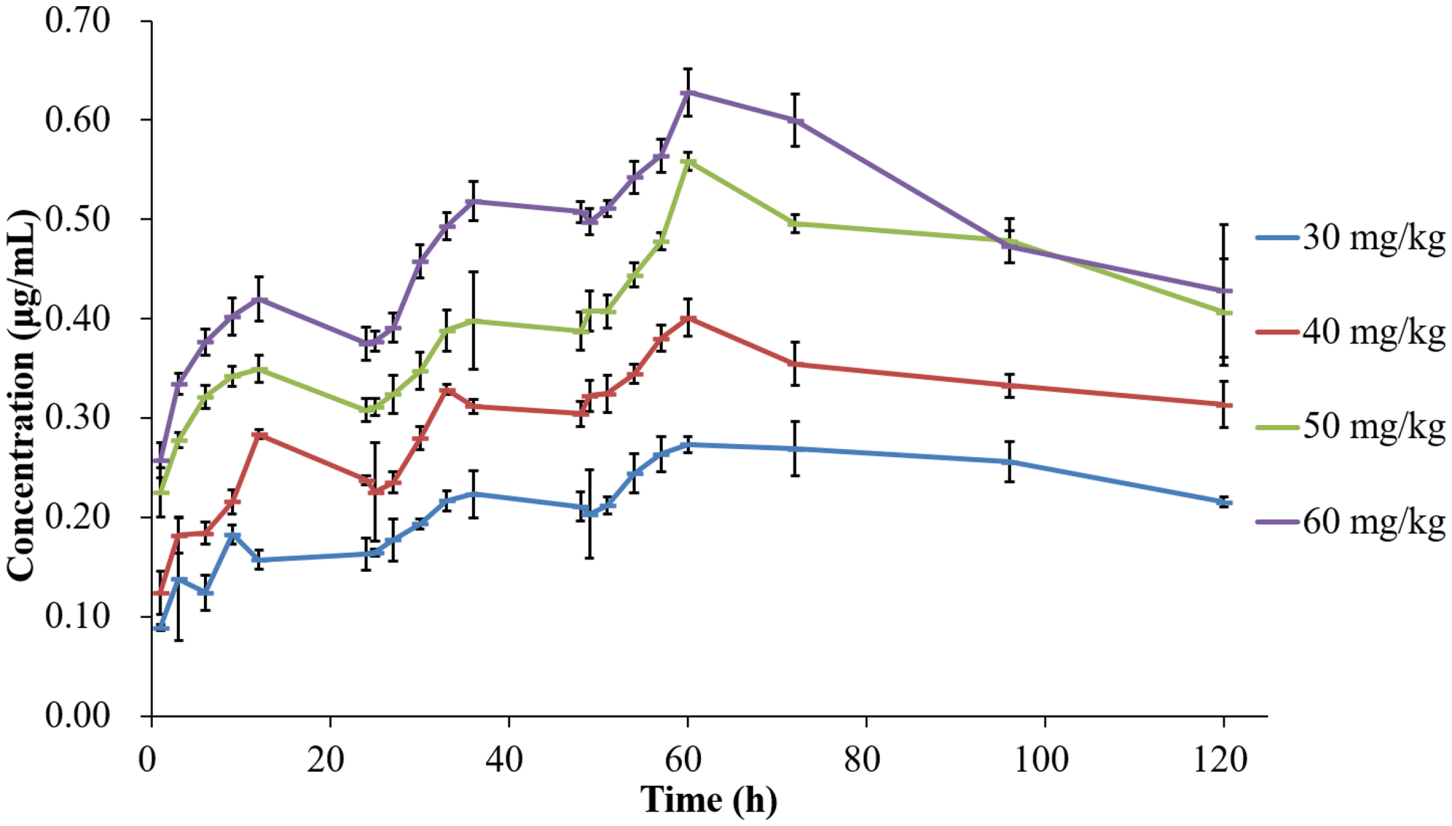
Concentration-time curves of tilmicosin in infected tissue cages following different multiple dosage administration.

The concentration-time profiles of tilmicosin in TCF following multiple dosing are depicted in Figure 1. The mean values for AUC_24h_, C_max_, and %T > MIC are presented in Table 1. Pharmacokinetic analysis was performed using non-compartmental methods in WinNonlin software. The determined AUC_24h_ ranged from 3.57 ± 0.20 to 13.65 ± 0.38 μg h/mL. C_max_ values were obtained directly from the concentration-time curves, spanning 0.20 ± 0.03 to 0.63 ± 0.03 μg/mL. The %T > MIC values covered a range of 0 to 100%. Specifically, the AUC values from 24–48 h after the final dose at 30, 40, 50 and 60 mg/kg were 6.31 ± 0.51, 8.35 ± 0.36, 11.69 ± 0.33, and 12.87 ± 0.22 μg h/mL, respectively.

**Table 1:** Pharmacokinetic parameters of tilmicosin in infected tissue cage fluid after multiple administration.

Dosage (mg/kg)	Dosing times	AUC_0-24h_ (μg·h/mL)	C_max_ (μg/mL)	T>MIC (h)
30	1	3.57 ± 0.20	0.20 ± 0.03	0.00 ± 0.00
	2	4.87 ± 0.34	0.23 ± 0.02	0.75 ± 1.49
	3	6.03 ± 0.14	0.29 ± 0.01	16.14 ± 1.58
40	1	5.40 ± 0.07	0.28 ± 0.01	10.42 ± 1.18
	2	6.92 ± 0.17	0.33 ± 0.01	20.74 ± 1.72
	3	8.61 ± 0.27	0.40 ± 0.02	24.00 ± 0.00
50	1	7.49 ± 0.15	0.36 ± 0.01	22.40 ± 1.09
	2	10.47 ± 0.60	0.42 ± 0.01	24.00 ± 0.00
	3	12.30 ± 0.11	0.56 ± 0.01	24.00 ± 0.00
60	1	8.96 ± 0.23	0.43 ± 0.01	23.66 ± 0.68
	2	11.33 ± 0.18	0.52 ± 0.02	24.00 ± 0.00
	3	13.65 ± 0.38	0.63 ± 0.03	24.00 ± 0.00

AUC_24h_, area under the concentration-time curve from 0 to 24 h after every administration; C_max_, the maximum concentration during the dosage interval; T > MIC, the time of drug concentration remains above the MIC. The values shown are means ± standard deviations.

### 3.3. *In vivo* antibacterial studies tilmicosin against *Pasteurella multocida*

**Figure 2:**
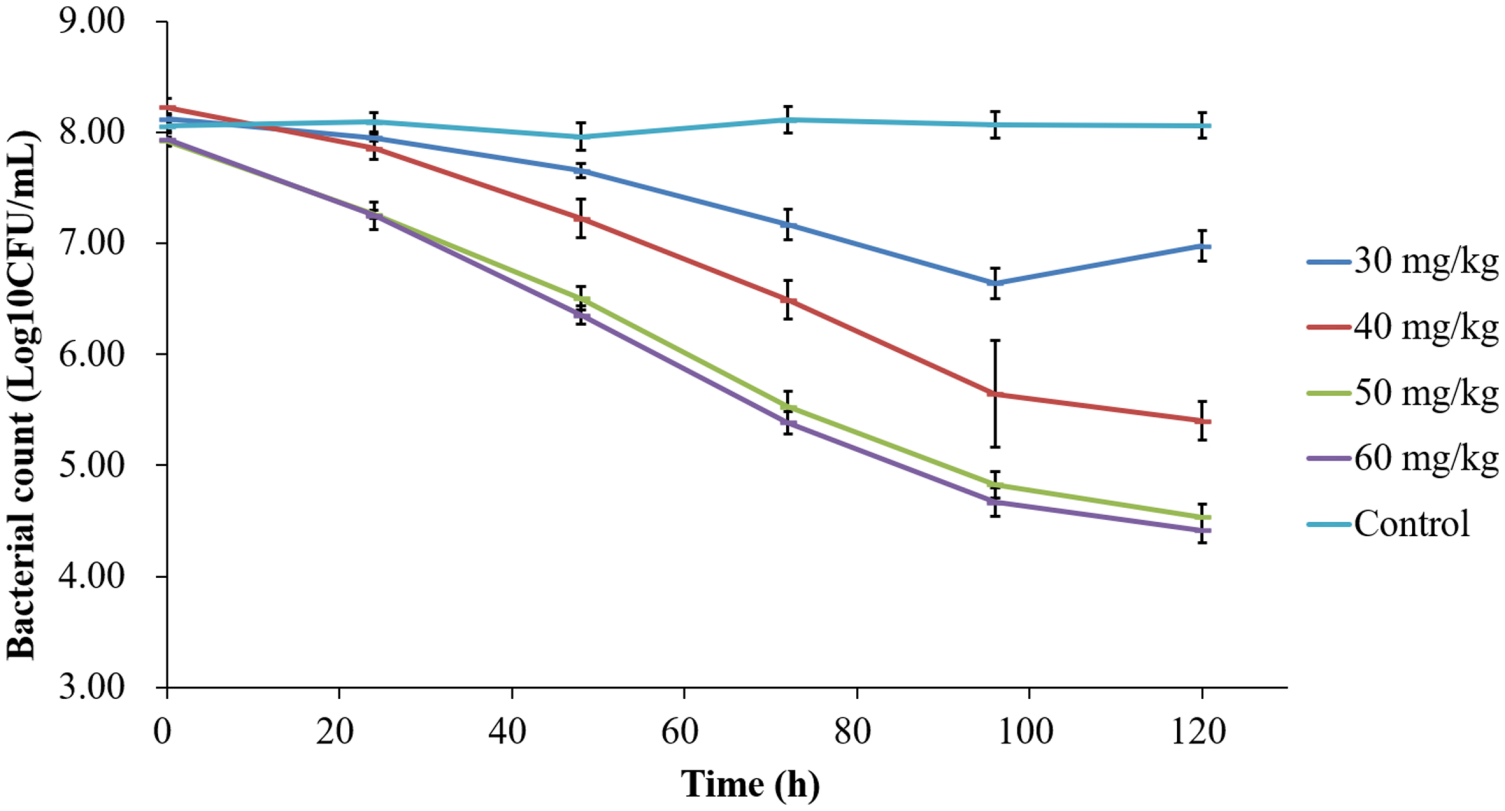
Time-killing curves of tilmicosin against *P. multocida* at different dosages in the tissue-cage model.

The time-kill curves depicted in Figure 2 demonstrate the antibacterial efficacy of tilmicosin against *P. multocida* in the piglet tissue-cage infection model across varying doses. Bacterial counts for the untreated control group remained around 10^7^ CFU/mL. Following tilmicosin administration at 30, 40, 50, and 60 mg/kg for 3 days, the total reductions in bacteria (log_10_ CFU/mL) were 1.48 ± 0.13, 2.82 ± 0.10, 3.39 ± 0.11, and 3.52 ± 0.15, respectively. Tilmicosin exhibited bactericidal activity at doses of 50 and 60 mg/kg after multiple administrations.

### 3.4. *In vivo* pharmacokinetic/pharmacodynamic integration and modeling

**Figure 3:**
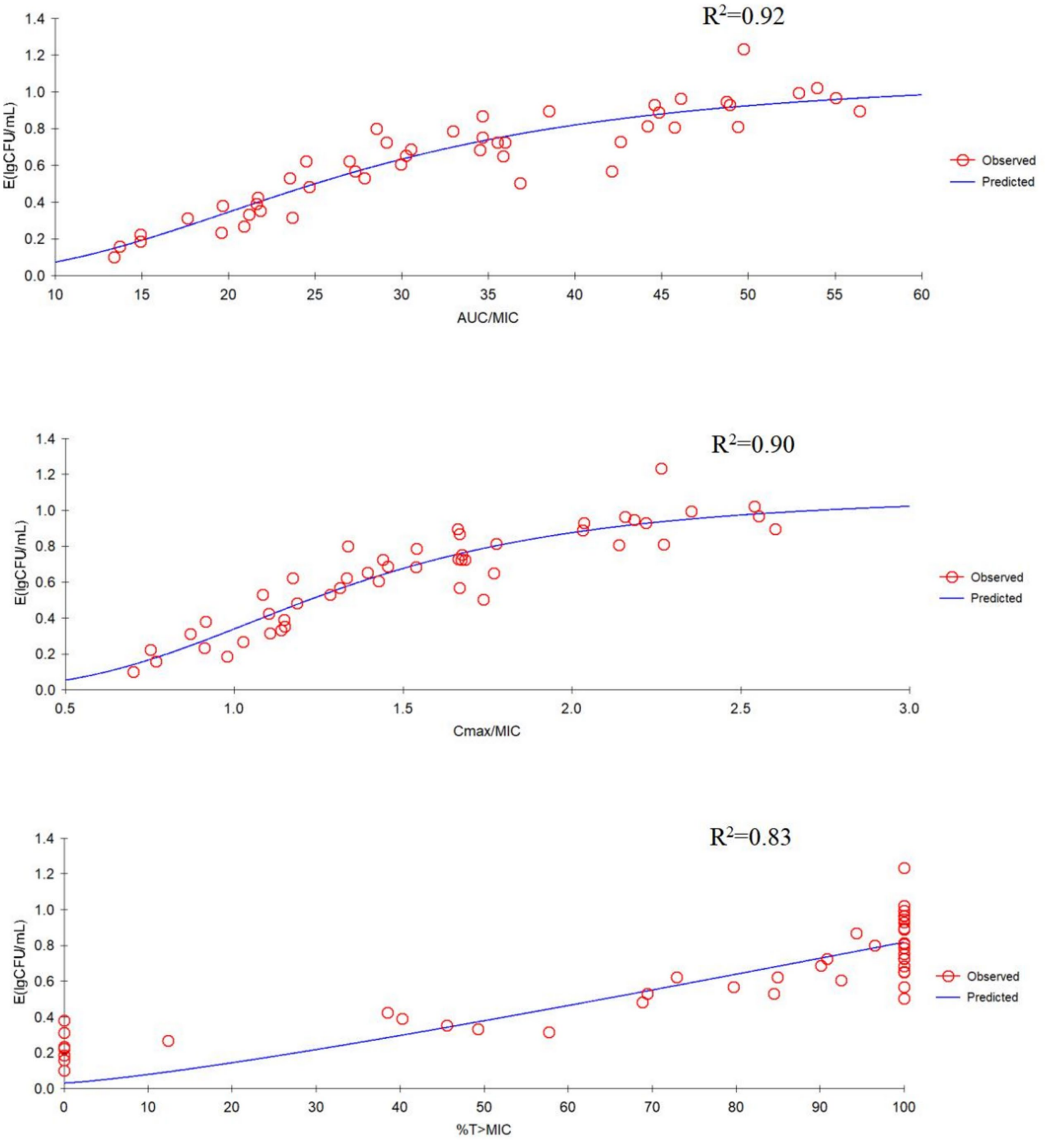
E_max_ relationships for the three pharmacokinetic/pharmacodynamic (PK/PD) parameters versus antibacterial effect.

The correlation between three PK/PD parameters (AUC_24h_/MIC, C_max_/MIC, and %T > MIC) and antibacterial activity of tilmicosin against *P. multocida* in TCF derived from Sigmoid E_max_ model was shown in Figure 3. The R^2^ of AUC_24h_/MIC, C_max_/MIC, and %T > MIC with an antibacterial effect was 0.92, 0.90, and 0.83, respectively. The obtained parameters of E_0_, E_max_, EC_50_, and the Hill coefficient are listed in Table 2. The estimated AUC_24h_/MIC values for a 1/3 log_10_CFU/mL, 1/2 log_10_CFU/mL, and 3/4 log_10_CFU/mL reduction were 19.64 h, 33.45 h, and 35.64 h, respectively, during the 24 h administration of tilmicosin. Figure 3 illustrates the correlation between three PK/PD indices (AUC_24h_/MIC, C_max_/MIC, and %T > MIC) and the antibacterial activity of tilmicosin against *P. multocida* in tissue cage fluid, as modeled using the Sigmoid E_max_ model. The coefficients of determination (R^2^) for AUC_24h_/MIC, C_max_/MIC, and %T > MIC with antibacterial effect were 0.92, 0.90, and 0.83, respectively. The derived E_0_, E_max_, EC_50_, and Hill coefficient parameters are tabulated in Table 2. Based on the model, the estimated AUC_24h_/MIC values required for a 1/3 log_10_CFU/mL, 1/2 log_10_CFU/mL, and 3/4 log_10_CFU/mL bacterial reduction over 24 h were 19.64 h, 33.45 h, and 35.64 h, respectively.

**Table 2:** The values of PK/PD parameters and AUC_24h_/MIC required for the indicated antibacterial effects.

Parameters	Values
*E_max_* (△log_10_ CFU/mL)	1.09
*E_0_* (△log_10_ CFU/mL)	0.003
*EC_50_*(h)	26.66
AUC_0-24h_/MICfor 1/3-log_10_ drop (h)	19.64
AUC_0-24h_/MIC for 1/2-log_10_ drop (h)	33.45
AUC_0-24h_/MIC for 3/4-log_10_ drop (h)	35.64
Slope (*N*)	2.69

## 4. Discussion

*P. multocida* is one of the most common bacterial species among porcine respiratory diseases, which greatly affects the production and health of swine and results in high economic losses. PK/PD modeling provides a chance for dosage optimization of antimicrobial therapy. Tissue cage model has been used to study the PK/PD integration of tulathromycin, marbofloxacin, and cefquinome on *P. multicida* (14, 15, 17). The dosage of other drugs commonly used in veterinary clinic, such as danofloxacin, florfenicol, ceftiofur, and oxytetracycline, have also been studied using PK/PD model (18–21). AUC_24h_/MIC was recognized as an important PK/PD parameter of macrolide antibiotic tulathromycin for antibacterial efficiency (14). As a semi-synthetic macrolide antibiotic with broad-spectrum activity, tilmicosin demonstrates potent efficacy against respiratory tract pathogens including *Actinobacillus pleuropneumoniae*, *Haemophilus parasuis*, and even *Mycoplasma pneumonia* (3, 22, 23). The study on the effect of tilmicosin on *P. multocida* will be helpful to the treatment of respiratory infection in pigs.

There may be differences in the antibacterial effect in artificial culture medium or in serum or tissue fluid. The MICs of tiamulin against several strains of *Actinobacillus pleuropneumoniae* in culture medium and pig serum were 12 and 24, 14 and 24, 12 and 32, and 12 and 24 μg/mL, respectively (24). Marbofloxacin has the same antibacterial effect on *Mannheimia haemolytica* in broth medium and sheep serum, MIC 0.035 μg/mL and MBC 0.045 μg/mL (25). For cefquinome against *P. multocida*, the MIC was similar in broth medium and body fluid, 0.03 μg/mL in medium and 0.04 μg/mL both in serum and tissue fluid (26). The MICs of tulathromycin against *P. multocida* CVCC430 in serum, transudate, and exudate were all found to be 0.03 μg/mL whereas in MHB medium it was 0.25 μg/mL, and the concentrations of tulathromycin in transudate and exudate were lower than those in serum (14). The determination of MIC in different media is necessary to study the antibacterial activity of drugs. In this study, the MICs of tilmicosin against *P. multocida* (D:7) in both TCF and TSB were 0.25 μg/mL, which was also used for *in vivo* PK/PD analysis. In this study, the data derived from tissue fluid in tissue cage model can better integrate pharmacokinetics and pharmacodynamics.

Oral administration of tilmicosin was adopted in this study. Study indicated subcutaneous injection of tilmicosin caused inflammation at the injection site (27, 28), and the side effects such as accelerated respiration, vomiting, convulsions, or even death may be produced after its intravenous and intramuscular administration or repeated high-dose administration (29). The potential application for an injectable formulation of tilmicosin for the treatment or control of porcine respiratory disease was contraindicated due to a low margin of safety. In clinical practice, the mixed feeding administration of tilmicosin at 200–400 mg/kg demonstrated a good effect on the prevention and treatment of pneumonia caused by *Actinobacillus pleuropneumoniae* and *P. multocida* (30, 31). Previous studies have found that most macrolide drugs exhibited time-dependent antibacterial activity, and two PK/PD parameters used to illustrate the antibacterial effect are AUC_24h_/MIC and %T > MIC. Before the present study, we had a preliminary study to design dose regimen. When the dose is below 20 mg/kg, the drug concentration is always lower than MIC. The ideal PK/PD parameters of T > MIC cannot be obtained, so the dose range for this study is 30 mg/kg to 60 mg/kg.

It is sometimes confusing to select the best PK/PD parameters to evaluate the efficacy of drugs based on the antibacterial characteristics (time-dependent or concentration-dependent), because the characteristics of the drug itself have a lot to do with it. The classification of drugs is not the only reference for selecting parameters to describe antibacterial effect. For drugs with long elimination half-life, PK/PD parameter AUC_24h_/MIC is usually appropriate to describe the effect. The t_1/2β_ of tilmicosin likely enables T > MIC to adequately suppress regrowth of the predominant bacterial population. This suggests that the AUC_24h_/MIC is the optimal PK/PD index to predict antibacterial activity. Azithromycin, time-dependent, because of its long elimination half-life, the best parameter for predicting the efficacy is AUC/MIC (32). The PK/PD parameters describing the effect of florfenicol is AUC_24h_/MIC for animals with long elimination half-life and %T > MIC could also be adopted for animals with short elimination half-life (33, 34). *In vitro* time-kill assays demonstrated a concentration-dependent antibacterial effect of tilmicosin against *Haemophilus parasuis* (22). The effect of tilmicosin against *Lawsonia intracellularis* was concentration-dependent, thus, AUC/MIC was used to formulate the dosage regimen (35). In the present study, the correlation between the PK/PD index of AUC_24h_/MIC and antibacterial effect was the highest (*R*^2^ = 0.92), which may be due to the slow elimination of tilmicosin in pigs. This is also an important reason why it is inappropriate to use% T > MIC to guide the administration of tilmicosin.

The relationship between AUC_24h_/MIC and bacterial change was fitted by Sigmoid model, and the magnitude of AUC_24h_/MIC required for a 3/4 log_10_CFU/mL of bacterial killing during 24 h was 35.64 h. Bactericidal activity was defined as >3-log_10_ reduction in CFU/mL. In this study, tilmicosin was administered once a day for 3 days. When the dosage was >50 mg/kg, the AUC_24h_/MIC was still >35.64 h in the period of 24–48 h after the last administration due to the slow elimination of tilmicosin, that is, the number of bacteria could be reduced by 3 log_10_CFU/mL within 96 h after administration and bactericidal effects can be achieved. In this study, the counts of bacteria under 50 and 60 mg/kg were no significantly difference (Figure 3). The possible reason is that for concentration-dependent drugs, the effect is enhanced with the increase of drug concentration within a certain range. When the dose is 50 mg/kg, the maximum effect has been achieved. Moreover, tilmicosin belongs to macrolide drugs, which are antibacterial drugs, and cannot completely eliminate the *P. multocida*.

Although some useful *in vivo* PK/PD data were obtained by establishing tissue cage infection model in the study, there are some limitations of this model. After all, the lung and not the tissue cage was the site of the infection. Compare with convention PK/PD model, physiological-based pharmacokinetic (PBPK) and hollow fiber infection model (HFIM) showed the power in flexibility and prediction. For instance, the concentrations under different doses of tilmicosin in different tissues even in pigs with different ages can be predicted by applying a PBPK model. In a HFIM, except the total counts of bacteria, the counts of resistant subpopulation and non-inherited resistant subpopulation (persistent cells and biofilms) can be detected. In future studies, PBPK model and HFIM model should be considered.

## 5. Conclusion

The current study investigated the PK/PD integration of tilmicosin against *P. multocida* strain D:7 was studied by establishing a tissue cage infection model in pigs. The results demonstrated that the AUC_24h_/MIC (*R*^2^ = 0.92) was the optimal PK/PD index for describing antibacterial activity. Further analysis indicated that an AUC_24h_/MIC value of 35.64 h was necessary for tilmicosin to achieve a 3/4 log_10_CFU/mL reduction in TCF. The bactericidal efficacy of tilmicosin against *P. multocida* was attained at doses of 50 mg/kg or higher, administered once daily for 3 days. These findings will facilitate guidance and optimization of clinical dosing regimens for treatment of *P. multocida* infections in piglets.

## Data availability statement

The original contributions presented in the study are included in the article/supplementary material, further inquiries can be directed to the corresponding author.

## Ethics statement

The animal study was approved by Committee on the Ethics of Animals of South China Agricultural University. The study was conducted in accordance with the local legislation and institutional requirements.

## Author contributions

YC: Writing – original draft, Data curation, Software. XJ: Validation, Writing – original draft. SZ: Validation, Writing – original draft. WW: Writing – review & editing. HZ: Writing – original draft. HD: Conceptualization, Funding acquisition, Project administration, Writing – original draft, Writing – review & editing.

## Funding

This work was supported by the National Natural Science Foundation of China (grant no. 31972733).

## Conflict of interest

The authors declare that the research was conducted in the absence of any commercial or financial relationships that could be construed as a potential conflict of interest.

## Publisher’s note

All claims expressed in this article are solely those of the authors and do not necessarily represent those of their affiliated organizations, or those of the publisher, the editors and the reviewers. Any product that may be evaluated in this article, or claim that may be made by its manufacturer, is not guaranteed or endorsed by the publisher.
